# Editor's choice: Tackling infectious diseases, NCDs and sexual reproductive health issues as we enter our 24^th^ year of remarkable growth

**DOI:** 10.4314/ahs.v24i1.1

**Published:** 2024-03

**Authors:** James K Tumwine

You are welcome to this first issue of 2024, which marks our entry into the 24^th^ year of publication of *African Health Sciences*. In August 2024, we shall be celebrating 24 years of steady growth and survival, as a journal in a highly competitive environment. We thank all our partners, authors, readers, reviewers, editorial team members, and our volunteer staff, and others, for your selfless, unconditional support. We do not take this support for granted, and we are extremely grateful to all of you. We are very proud of you as we, together, strive to push scientific publishing in Africa to greater heights. Thank you very much indeed!

In this March 2024 issue of *African Health Sciences*, we bring you papers on infectious diseases, sexual reproductive and child health, non-communicable diseases, and health system issues.

The eleven papers on infectious diseases are, as usual, dominated by HIV and tuberculosis^1^-^6^, and others^7^-^11^, such as viral hemorrhagic fever and otomycosis.

The next section covers sexual reproductive and child health^12^-^19^. These range from gender-based violence^12^-^13^, contraception^14^, screening for cervical cancer^15^, occupational hazards, preterm labour, and attitudes to abortion^16^-^19^.

The treatise on non-communicable diseases is somewhat on familiar territory covering diabetes mellitus^20^-^22^, cancer^23^; irritable bowel^24^, COPD^25^, Parkinson's disease^26^ and intussusception^27^.

The next section is a salad of sorts: surfactant, newborn screening, and nutrition status^28^-^30^. Health systems dominate the next section and include traditional health system referrals^31^-^32^, mobile phones in health care^33^-^34^, and an odd paper on deodrants in Palestine. The Editor's choice ends with two interesting papers on herbal medicine and pharmacogenetics.

We wish you very productive and enjoyable reading!
